# Molecular discrimination of *Opisthorchis*-like eggs from residents in a rural community of central Thailand

**DOI:** 10.1371/journal.pntd.0006030

**Published:** 2017-11-02

**Authors:** Saiwasan Buathong, Saovanee Leelayoova, Mathirut Mungthin, Toon Ruang-areerate, Tawee Naaglor, Picha Suwannahitatorn, Phunlerd Piyaraj, Paanjit Taamasri, Peerapan Tan-ariya

**Affiliations:** 1 Department of Microbiology, Faculty of Science, Mahidol University, Bangkok, Thailand; 2 Department of Parasitology, Phramongkutklao College of Medicine, Ratchathewi, Bangkok, Thailand; Istituto Superiore di Sanità, UNITED STATES

## Abstract

**Background:**

*Opisthorchis viverrini* infection is a major public health problem in northern and northeastern Thailand. The chronic infection of *O*. *viverrini* is related to cholangiocarcinoma which causes high mortality in endemic areas. Therefore, the diagnosis, treatment, control and prevention of *O*. *viverrini* infection are necessary. The morphology of the egg is very similar to that of other species of human liver flukes (*Opisthorchis felineus* and *Clonorchis sinensis*) as well as that of small intestinal flukes in the family Heterophyidae. Thus, molecular characterization is crucially required to discriminate species of *Opisthorchis*-like eggs in fecal examination.

**Methodology/Principal findings:**

We aimed to determine the prevalence of *O*. *viverrini* infection among villagers living in Sanamchaikate District, Chachoengsao Province, in central Thailand, where *O*. *viverrini* infection has previously been reported. A total of 2,609 fecal samples were examined for *Opisthorchis*-like eggs using microscopic examination. PCR-RFLP analysis of the ITS2 region was used to discriminate *Opisthorchis-*like eggs. The genetic structure of *O*. *viverrini* infection was demonstrated using nucleotide sequencing of cytochrome c oxidase subunit I (*cox1*) and NADH dehydrogenase subunit 1 (*nad1*). Testing of evolutionary neutrality of the *cox1* and *nad1* sequences of *O*. *viverrini* was performed using Tajima's *D* tests and Fu's *Fs* tests. Moreover, the haplotype networks and phylogenetic trees were constructed to study the relationships of *O*. *viverrini* isolated from different endemic areas. A high prevalence of *O*. *viverrini* infection is still observed in a rural community of Chachoengsao Province, central Thailand. The overall prevalence of *Opisthorchis*-like eggs using microscopic examination was 16.8%. PCR-RFLP profiles showed the predominant infection of *O*. *viverrini* (9.6%) including very low infections of other small intestinal flukes, *Haplorchis taichui* (0.08%) and *Euparyphium albuferensis* (0.08%). The genetic structure of *O*. *viverrini* populations in central Thailand was also described and revealed a non-significant difference in genetic diversity. In addition, the genetic background of the *O*. *viverrini* populations was closely related to the isolate from Lao PDR.

**Conclusions/Significance:**

Our study highlighted the prevalence of *O*. *viverrini* infection in central Thailand indicating that control programs and health education regarding opisthorchiasis is still required in this endemic area. Additionally, the study demonstrated the genetic structure of *O*. *viverrini*, in central Thailand which could provide information on the molecular epidemiology of this parasite.

## Author summary

*O*. *viverrini* infection is highly prevalent in northern and northeastern Thailand. The diagnosis of the infection is usually achieved by finding the eggs in feces. However, these eggs are difficult to differentiate morphologically from other *Opisthorchis*-like eggs. Our study evaluated the prevalence and molecular characterization of *Opisthorchis*-like eggs in fecal samples collected from 2,609 villagers living in a rural community, in central Thailand, using PCR-RFLP analysis of ITS2 region. This study insists that Sanamchaikate District, Chachoengsao Province, central area, is one of the areas for *O*. *viverrini* infection. To understand the transmission dynamics of *O*. *viverrini* in the study area, the genetic structure of *O*. *viverrini* was also assessed using *cox1* and *nad1* sequences. The *O*. *viverrini* populations showed monophyly and the genetic background was closely related to one isolate from Lao PDR.

## Introduction

*Opisthorchis viverrini*, a fish-borne trematode (FBT), is one of the main public health problems in Southeast Asia, while *Clonorchis sinensis* is commonly found in Japan, the Republic of Korea, China, Taiwan and Vietnam [1,2]. In Thailand, more than 3.3 million people are predominantly infected with *O*. *viverrini*, the distribution of which varies depending on endemic areas [3]. Infection with *O*. *viverrini* is often asymptomatic, while heavy infection may lead to obstructive jaundice. Other symptoms could occur, including relapsing cholangitis, periductal fibrosis, cholecystitis and/or cholelithiasis [4,5]. The most severe sequelae of *O*. *viverrini* and *C*. *sinensis* infections, is cholangiocarcinoma (CCA), of which the highest incidence of 93.8–317.6 per 100,000 person-years has been reported in northeastern Thailand [6–9]. Currently, the carcinogenic potential of *Opisthorchis felineus* has been reported, and further investigation into the compelling evidence that *O*. *felineus* infection could cause CCA is needed [10–12]. The gold standard for diagnosis of liver fluke infection is the formalin-ethyl acetate concentration technique (FECT) [13,14]; moreover, enzyme-linked immunosorbent assay (ELISA) [15–18] and molecular techniques [19–29] were also developed to detect the infection. Since *O*. *viverrini* and small intestinal flukes; such as *Haplorchis taichui*, *Haplorchis pumilio*, *Haplorchis yogokawai*, *Heterophyes heterophyes* and other usually share the same endemic area and the route of transmission, co-infections are commonly found. [30]. The eggs of *O*. *viverrini* and those of small intestinal flukes are very similar in size and shape and cannot be clearly discriminated under light microscope. Thus, these eggs are identified as *Opisthorchis*-like eggs for which a molecular technique is required for genus and species identification [31,32]. Specific primers or DNA probes have been developed to detect *O*. *viverrini* eggs in fecal samples as well as several larval stages of *O*. *viverrini* in *Bithynia* snails and metacercariae in cyprinoid fish [19–29,33,34]. Among several genetic markers used to differentiate *Opisthorchis*-like eggs, the internal transcribe spacer 1 (ITS1) and internal transcribe spacer 2 (ITS2) of the ribosomal RNA (rRNA) genes have been recently used as markers to discriminate *O*. *viverrini*, *C*. *sinensis*, and small intestinal flukes [23,24]. Moreover, the mitochondrial gene, cytochrome c oxidase subunit 1 (*cox1*), NADH dehydrogenase subunit 1 (*nad1*) and NADH dehydrogenase subunit 2 (*nad2*), have also been used to discriminate *O*. *viverrini* from *C*. *sinensis* and *H*. *taichui* [22,26,35]. Recently, genetic diversity on species complex of *O*. *viverrini* has been found in different geographical areas in Thailand and Lao PDR using multilocus enzyme electrophoresis analyses (MEE) [36,37]. Analyses include random amplified polymorphic DNA (RAPD) [38], mitochondrial DNA sequencing [39,40] and microsatellite marker analysis [41]. Using the mitochondrial *nad1* and *cox1* sequences, different haplotypes were found among *O*. *viverrini* isolates in northeastern Thailand, as well as in Lao PDR [39]. In contrast, no genetic diversity of *O*. *viverrini* was found among isolates of *O*. *viverrini* from Thailand, Cambodia and Lao PDR using *nad1* sequence analysis [40].

Apart from northern and northeastern Thailand, rural communities of central Thailand including in Sanamchaikate District, Chachoengsao Province, have been shown to be the endemic areas of *O*. *viverrini* infection in central Thailand [42,43]. Due to the migration of local people from northeastern provinces now living in this community, life styles as well as the habit of consuming undercooked or raw freshwater fish have not changed. Related studies have shown a higher prevalence of 21.3% in 2009 [42] and 24.0% in 2013 [43] compared with the average prevalence of 3.8% reported in central Thailand [44]. In addition, Traub et al. first reported *C*. *sinensis* infection among Thai villagers living in this community [24]. Thus, we aimed to investigate the prevalence of *O*. *viverrini* and other FBT infections in fecal specimens from people living in three villages located in Sanamchaikate District, Chachoengsao Province. The species of *Opisthorchis*-like eggs were determined using PCR-RFLP analysis of ITS2 region. Genetic diversity and the population structure of *O*. *viverrini* infection have been studied in northeast Thailand, except for those in this study area. Studying the genetic background of *O*. *viverrini* would help to understand the molecular epidemiology and transmission dynamics of the parasite. Thus, we first described the genetic background of *O*. *viverrini* infection in a rural community, central Thailand, using DNA sequence analysis of the mitochondrial genes, *cox1* and *nad1*.

## Materials and methods

### Study area

A cross-sectional study was conducted in a rural community, Sanamchaikate District, Chachoengsao Province, Central Thailand, 228 km east of Bangkok. Villagers from the three villages were enrolled in the study, that is, Na-Ngam, Thoong-Heang and Na-Yao Villages. Proper anthelminthic treatment and health education were provided for those who had intestinal parasitic infections. *Opisthorchis-*like egg–infected cases were treated with a single dose of praziquantel (40 mg/kg).

### Ethics approval and consent to participate

The research protocol was reviewed and approved by the Ethics Committee of The Royal Thai Army Medical Department (Ref. S025h/51 and S045h/54) and Mahidol University Institutional Review Board (MU-IRB) (Ref. MU-IRB 2014/018.0108). Written informed consent was obtained from the enrolled participants or parents of young participants using standard protocols approved by the Ethics Committee of the Royal Thai Army Medical Department, Thailand.

### Fecal sample collection

A total of 2,609 fecal samples were collected from participants living in three villages. Of these, 689 samples from Thoong-Heang and 858 from Na-Ngam villagers were collected in February and September 2008, respectively with the remaining 1,062 samples from Na-Yao villagers being collected in February 2013. Fecal samples were examined for *Opisthorchis*-like eggs using simple wet smear, Kato thick smear method and phosphate buffer saline (PBS) ethyl acetate concentration technique. *Opisthorchis* like-eggs positivity was defined as the presence of the eggs in the fecal specimen examined by at least one of the three methods.

### DNA extraction of *Opisthorchis*-like eggs

*Opisthorchis*-like eggs collected from PBS ethyl acetate sediments were used for DNA extraction. A 200 μl aliquot of each positive sample was treated with 1.4 mL ATL tissue lysis buffer (Qiagen), mixed continuously for 1 min or until the stool samples were thoroughly homogenized. The suspension was subjected to PBS incubation technique [28] and 5 cycles of freezing in liquid nitrogen followed by thawing at 98°C to 100°C [24] to break up the eggs. Subsequently, the suspension was heated at 70°C for 5 min before continuously mixing and centrifuging at 20,000 *g* for 1 min to sediment fecal pellets. Then 1.2 mL of supernatant was transferred into a new 2 mL microcentrifuge tube. The DNA was extracted from the supernatant using the QIAmp DNA stool mini kit (Qiagen) according to the manufacturer’s protocol. At the final step, DNA was eluted with 50 μl of elution buffer. At the final step, DNA was eluted with 50 μl of elution buffer. DNA extraction of the positive control (*O*. *viverrini* eggs) was also performed and doubled distilled water was used as negative control. Other negative fecal samples using wet smears, Kato thick smear and PBS ethyl acetate concentration technique were not used for the PCR assay.

### PCR and PCR-restriction fragment length polymorphism (PCR-RFLP) methods for discriminating eggs of *O*. *viverrini*, *C*. *sinensis* and small intestinal flukes

The RTFluke primers designed for ITS2 region of opisthorchiid and heterophyid flukes, were used to amplify DNA extracts of the eggs [24]. PCR amplifications were carried out in a final volume of 50 μl, consisting of DNA template, 12.5 pmol of RTFlukeFa; 5'- CTTGAACGCACATTGCGGCC-3’ and RTFlukeRa; 5'-CACGTTTGAGCCGAGGTCAG-3', 200 μM dNTP, 2 mM of MgCl_2_, 1X buffer PCR and 1 unit of *Taq* polymerase (5U/μl) (Promega). The PCR products were amplified in the Mastercycle personal (Bio-Rad). The PCR assay consisted of an initial stage of denaturation 94°C for 15 min, annealing temperature at 60°C for 1 min, extension step at 72°C for 2 min followed by 35 cycles of 94°C for 30 sec, 60°C for 30 sec, 72°C for 30 sec, a final extension at 72°C for 7 min and a holding temperature of 12°C to complete the amplification. The amplicons of *O*. *viverrini*, *C*. *sinensis* and *H*. *taichui* were 375, 381 and 526 bp, respectively [24].

The PCR products at 375 bp and 381 bp were subjected to PCR-RFLP while PCR products of 526 bp were sent for DNA sequencing. PCR-RFLP was performed to discriminate eggs of *O*. *viverrini* and *C*. *sinensis* using a restriction enzyme, *Fau*I. PCR products were digested with 2 units of *Fau*I (New England Biolabs) in a total volume of 20 μl at 55°C for 6 to 8 h.

### Genetic diversity and genetic structure of *O*. *viverrini* using the cytochrome c oxidase subunit 1 gene (*cox1*) and the NADH dehydrogenase subunit 1 gene (*nad1*)

To study the genetic diversity of *O*. *viverrini* eggs, the PCR assays were performed according to Bauthong et al., 2005 [28]. The primers, COXI-OvF; 5′-TGATCCGTTGTTGTTTCA-3′ and COXI-OvR; 5′-ACGGATATAACCACCGTTCT-3′) were used for *cox1*. The primers for *nad1* were NADI-OvF; 5′-TGTTGAAGATGATTGAG-3′ and NADI-OvR; 5′-CAAGGTTAACCTAACGA-3′, respectively. The PCR assay was performed in a total volume of 50 μl, consisting of DNA template, 25 pmol of each primer, 200 μM dNTP, 2 mM of MgCl_2_, 1X buffer, 1X enhancer, and 1 unit of KAPA2G Robust Hotstart polymerase (5U/μl) (KAPA Biosystems). The PCR products were amplified in the Mastercycle personal (Bio-Rad). The PCR assay of COXI-Ov primer was initiated at predenaturation at 95°C for 5 min, followed by 30 cycles of denaturation at 95°C for 30 sec, annealing at 58°C for 30 sec, extension at 72°C for 30 sec, final extension was 72°C for 7 min and the holding temperature at 12°C to complete amplification. For NADI-Ov primer, the predenaturation temperature was 95°C for 5 min followed by denaturation at 95°C for 30 sec, the annealing temperature at 55°C for 30 sec, extension step at 72°C for 30 sec, final extension at 72°C for 7 min, and the holding temperature at 12°C [28]. The PCR products were run on a 2% agarose gel in 1X Tris/borate/EDTA (TBE) buffer. For DNA staining, 10 mL of agarose gel was mixed with 0.3 μl SYBR safe DNA Gel Stain (Invitrogen). A 100 bp DNA ladder (Vivantis Technologies) was used as the marker to estimate the sizes of PCR products. The PCR products were run at 100 V at room temperature for 40 min. Finally, the agarose gel was visualized by molecular image Gel doc XR+ Imaging System (Bio-Rad). The PCR products were purified before DNA sequencing using QIAquick Gel extraction kit (Qiagen) according to the manufacturer’s protocol. All PCR products were sent to the First BASE laboratories (First BASE Laboratories) for DNA sequencing. The sequences were subjected to NCBI BLAST search to examine nucleotide sequences and identify species [45]. The DNA sequences were aligned using BioEdit software, version 7.0.9 to observe similarities [46].

### Population genetic analysis of the *cox1* and *nad1* sequences

To study population genetics, a total of 90 ITS2-PCR positive samples were randomly selected from the three villages (30 samples from each village). Genetic analysis of the *cox1* and *nad1* of FTB was performed to assess haplotype diversity numbers (Hd), segregation sites between populations (S) and nucleotide diversity (Pi) using DnaSP, version 5.10.1. [47]. The *cox1* and *nad1* evolutionary neutrality of the DNA sequences were evaluated using Tajima's *D* tests [48], Fu's *Fs* test [49] and Arlequin, version 3.5.2.2 [50]. The statistical significance of neutrality tests including Tajima’s *D*, and Fu’s *Fs* to examine genetic hitchhiking, population expansion, selective sweep and bottleneck were determined at 95% interval (*p* <0.05). The haplotype networks were constructed using median-joining network from the Network 5.0.0.0 program, which is available at http://www.fluxus-engineering.com.

### Phylogenetic trees of the *cox1* and *nad1* of *O*. *viverrini* and other trematodes

To generate a phylogenetic tree, the *cox1* and *nad1* sequences were separately aligned with reference *cox1* and *nad1* sequences of *O*. *viverrini* using ClustalW program in BioEdit software, version 7.1.9 [46]. The reference sequences of *cox1* and *nad1* of *O*. *viverrini* with *C*. *sinensis* as an outgroup, were retrieved from GenBank for phylogenetic tree analysis. The Randomized Axelerated Maximum Likelihood (RAxML) trees of different genes, *cox1* and *nad1*, were constructed based on RAxML version 7.4.2 with a GTR matrix (GTR + Γ model) [51] using raxmlGUI version 1.3 [52]. The clade stability of the branding topologies of *cox1* and *nad1* sequences was evaluated using 1,000 replicates of RAxML bootstrap values.

### Accession numbers

All data are available through the NCBI data base with the following accession numbers, *cox1* sequences: EU022353.1, EU022351.1, EU022354.1, EU022356.1, EU022355.1, EU022360.1, EU022364.1, EU022363.1, EU022362.1, EU022352.1, EU022357.1, EU022358.1, EU022359.1, JF739555.1 and JN936215.1 *nad1* sequences: EU022338.1, EU022344.1, EU022349.1, EU022346.1, EU022345.1, EU022348.1, EU022350.1, EU022343.1, EU0222334.1, EU022337.1, EU022342.1, EU022339.1, EU022347.1, JF739555.1, DQ119551.1, EU022340.1, GQ401040.1, DQ882172.1, EU443831.1, DQ882175.1, DQ882173.1, EU443833.1, EU443832.1, DQ882174.1, GQ401082.1 and JF729304.1.

## Results

### The prevalence of *Opisthorchis*-like eggs and *O*. *viverrini*

**Fig 1:**
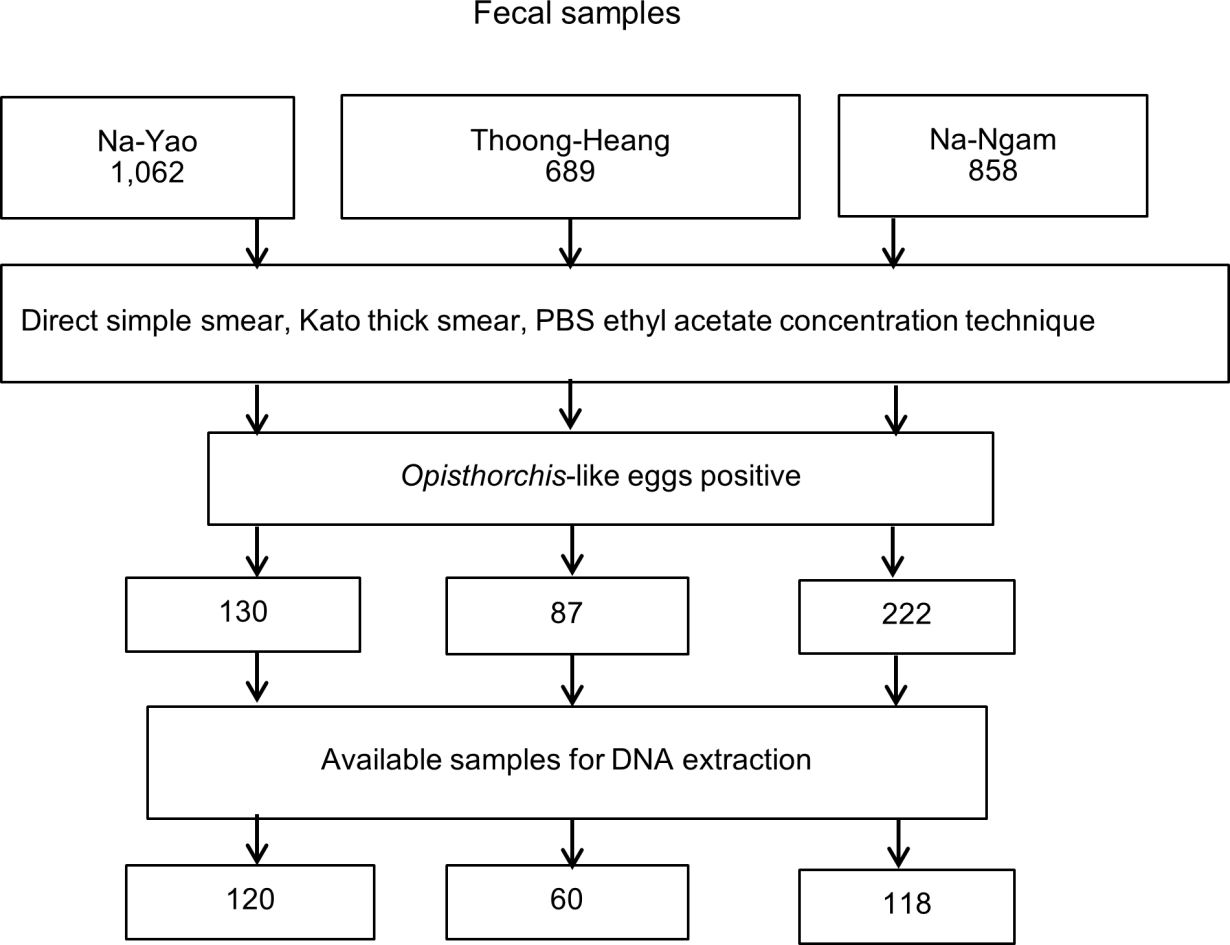
A flow diagram illustrating the methods used for fecal examination, *Opisthorchis*-like egg positive samples and available fecal samples for DNA extraction.

**Fig 2:**
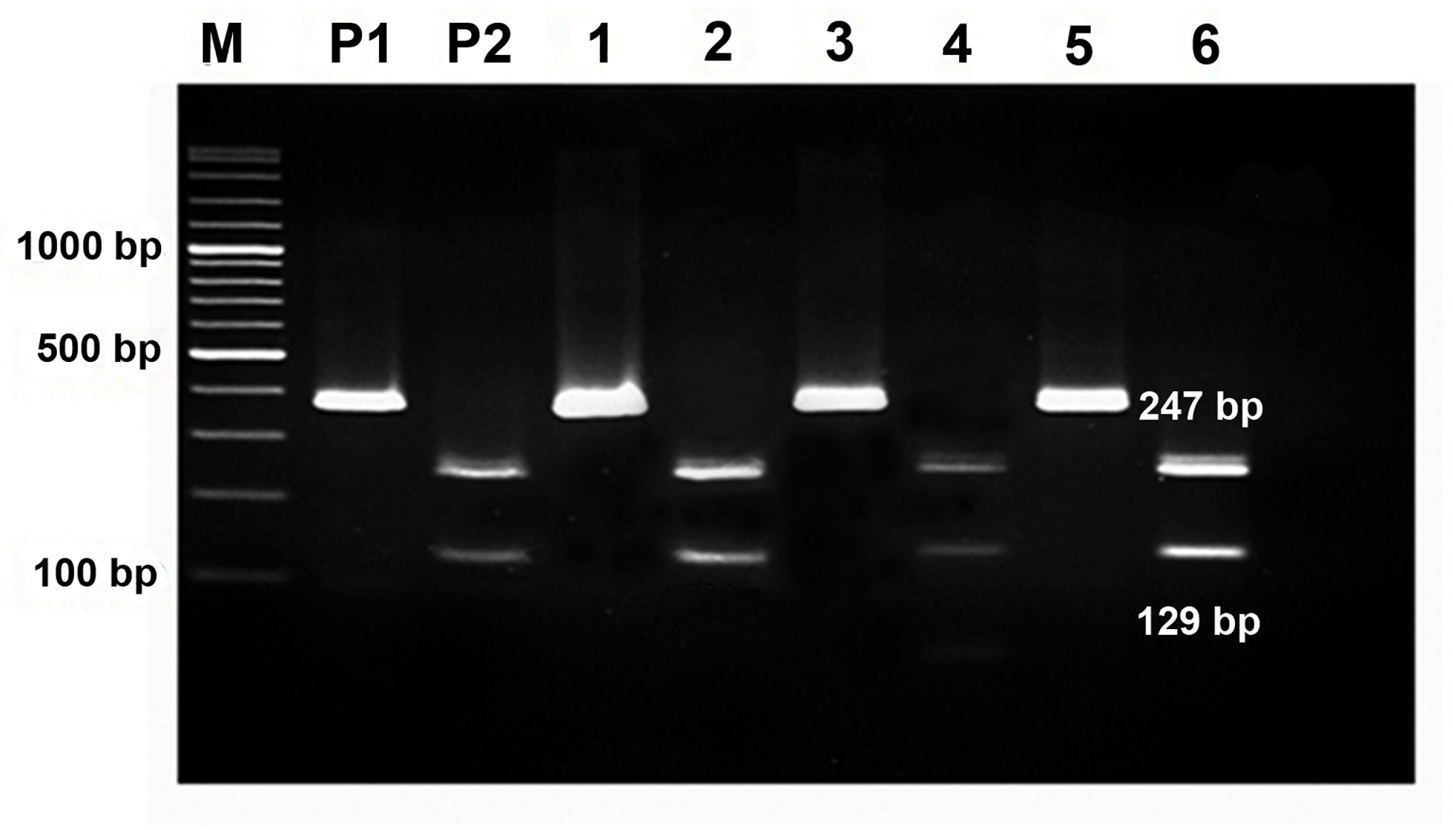
The PCR-RFLP products of *O*. *viverrini* after digestion with *Fua*I restriction enzyme. Amplicons of 247 bp and 129 bp from ITS2-PCR products of *O*. *viverrini* were generated after digestion. M was 100 bp DNA ladder, P1 was undigested positive and P2 was digested PCR products of positive samples. Numbers 1–6 showed undigested and digested ITS2-PCR products from fecal samples.

A total of 298 *Opisthorchis*-like egg positive fecal samples were available for DNA extraction and PCR assays (Fig 1). To identify species of *Opisthorchis*-like eggs, the ITS2-PCR assay gave specific amplicons of 375, 381 and 526 bp for *O*. *viverrini*, *C*. *sinensis* and *H*. *taichui*, respectively. The ITS2-PCR sensitivities and prevalence of *Opisthorchis*-like egg infection in Thoong-Heang, Na-Yao and Na-Ngam Villages are shown in Table 1. The PCR-RFLP products showed the fragments of 129 bp and 247 bp for *O*. *viverrini* but gave an undigested amplicon for *C*. *sinensis* (Fig 2). As shown in Table 1, of 253 ITS2-PCR samples, PCR-RFLP profiles of *O*. *viverrini* were as follows: 7.8% (54/689) from Thoong-Heang Village, 12.5% (107/858) from Na-Ngam Village and 8.3% (88/1,062) from Na-Yao Village. Mixed infection of *O*. *viverrini* and *H*. *taichui* was observed at 0.23% (2/858) in Na-Ngam Village and 0.2% (2/1,062) of *Euparyphium albuferensis* infection was detected in Na-Yao Village. Thus, the overall infection of *O*. *viverrini*, *H*. *taichui* and *E*. *albuferensis* was 9.6% (251/2,609), 0.08% (2/2,609) and 0.08% (2/2,609), respectively. The PCR products of 520 bp revealed *H*. *taichui* and *E*. *albuferensis*, which were confirmed by DNA sequencing and NCBI BLAST search.

**Table 1:** The prevalences of *Opisthorchis*-like egg, ITS2-PCR sensitivities and PCR-RFLP profiles for detection *Opisthorchis*-like eggs in Chachoengsao Province, central Thailand.

Village	Prevalence	ITS2-PCR sensitivity	Parasite identification by PCR-RFLP (Number)
Thoong-Heang	12.6% (87/689)	90.0% (54/60)	*O*. *viverrini* (54)
Na-Yao	12.2% (130/1,062)	75.0% (90/120)	*O*. *viverrini* (88) *E*. *albuferensis* (2)
Na-Ngam	25.9% (222/857)	92.4% (109/118)	*O*. *viverrini* (107) *O*. *viverrini* and *H*. *taichui* (2)
Total	16.8% (439/2,609)	84.9% (253/298)	

### Population genetic analysis using the *cox1* and *nad1* sequences of *O*. *viverrini*

The PCR products of mitochondrial *cox1* and *nad1* were 504 bp and 780 bp, respectively [28]. Of the 90 ITS2-PCR positive samples, 60 (66.7%) *cox1* and 45 (50.0%) *nad1* could be successfully amplified, bidirectionally sequenced and were used for population genetics analysis. Using the *cox1* sequences, *O*. *viverrini* isolated from Na-Yao Village revealed the highest genetic diversity while reference isolates showed the lowest genetic diversity. The results of neutrality testing for *O*. *viverrini* from the three villages did not significantly differ using both Fu's *Fs* and Tajima's *D* test (*p*>0.05) (Table 2).

**Table 2:** The indicators of genetic diversity and neutrality tests in the populations of *O*. *viverrini* from three villages using *cox1* sequences.

Populations	No. of samples	Haplotype	S	Genetic diversity		Neutrality tests	
				Hd	Pi	Tajima' s *D* (*p* value)	Fu's *Fs* (*p* value)
Na-Yao	19	12	28	0.871	0.031 +/ 0.016	1.418*	0.238*
Thoong-Heang	18	8	26	0.824	0.026 +/-0.013	0.810*	2.751*
Na-Ngam	19	8	9	0.614	0.009 +/-0.005	-0.359*	-0.515*
Reference isolates	15	3	5	0.514	0.003 +/- 0.002	-0.657*	1.412*
Total populations	69	29	55	0.892	0.024 +/- 0.012	-0.761*	-4.048*

S = no. of segregation sites (polymorphic), Hd = haplotype diversity, Pi = nucleotide diversity. Statistical significance: *p* < 0.05.

* *p*>0.05, not significantly different.

Regarding the *nad1* sequences of *O*. *viverrini*, the *nad1* sequences from those collected from reference isolates revealed the highest genetic diversity while the lowest genetic diversity was found among those of Thoong-Heang Village. Moreover, the neutrality tests of *nad1* did not significantly differ using both Fu's *Fs* and Tajima’s *D* test (*p*>0.05) (Table 3).

**Table 3:** The indicators of genetic diversity and neutrality tests in the populations of *O*. *viverrini* from three villages using *nad1* sequences.

Populations	No. of samples	Haplotype	S	Genetic diversity		Neutrality tests	
				Hd	Pi	Tajima' s *D* (*p* value)	Fu's *Fs* (*p* value)
Na-Yao	15	9	40	0.800	0.018 +/- 0.010	-0.266*	1.449*
Thoong-Heang	13	5	22	0.692	0.013+/-0.007	0.950*	4.579*
Na-Ngam	14	6	16	0.736	0.010 +/- 0.005	0.763*	2.085*
Referent isolates	25	13	13	0.917	0.012 +/-0.006	-0.456*	-0.538 *
Total populations	67	31	80	0.951	0.021 +/-0.010	-1.071*	-2.673*

S = no. of segregation sites (polymorphic), Hd = haplotype diversity, Pi = nucleotide diversity. Statistical significance: *p* < 0.05.

* *p*>0.05, not significantly different.

### Haplotype networks of *cox1* and *nad1* sequences in the populations of *O*. *viverrini*

**Fig 3:**
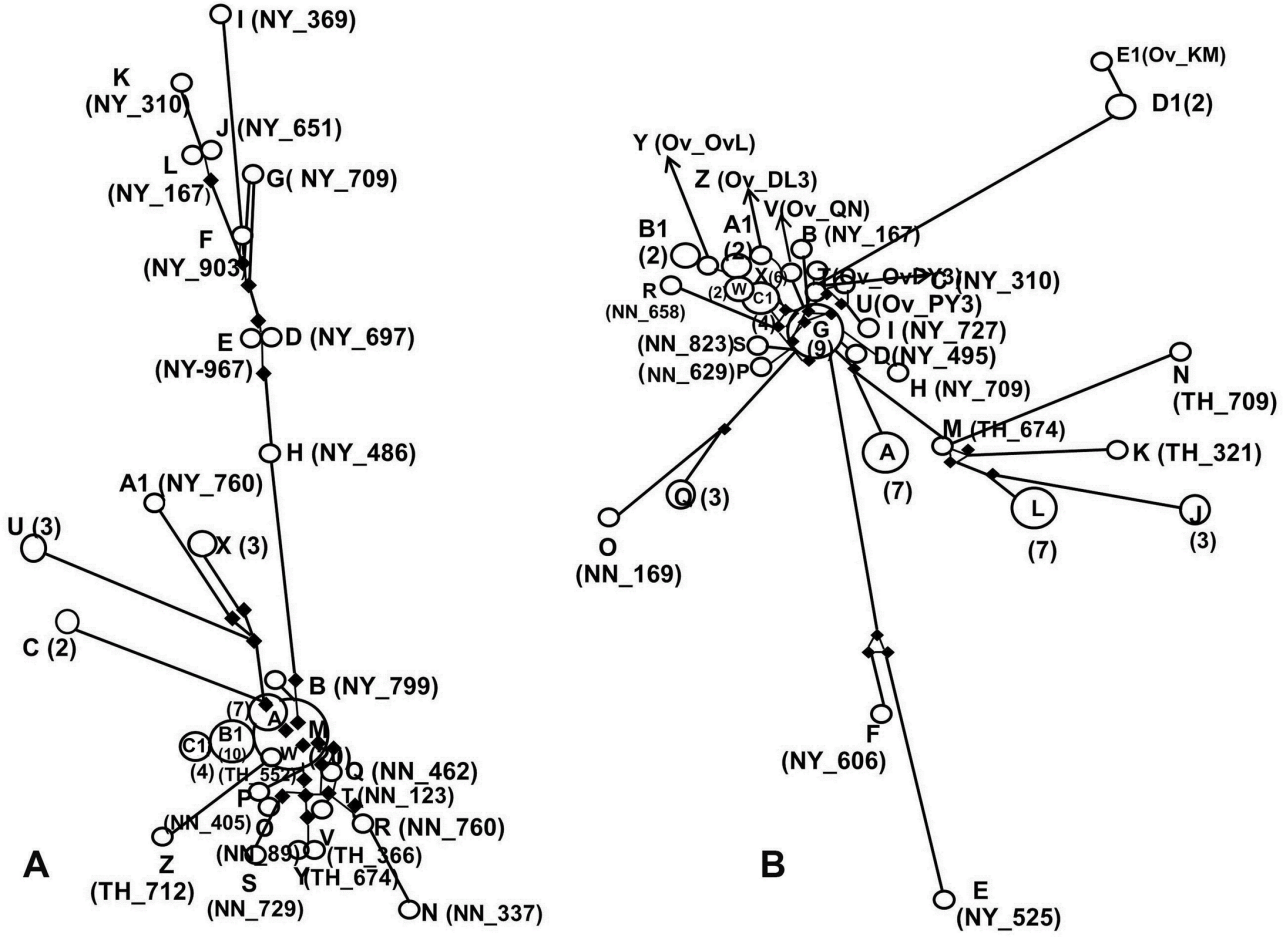
The haplotype networks of *cox1* and *nad1* sequences of *O*. *viverrini* isolated from three villages in central Thailand, reference isolates from Thailand, Lao PDR, Vietnam and Cambodia. (A) The haplotype network of 29 haplotypes based on *cox1* sequences. Haplotype M contains 20 isolates and shows highest frequency; seven isolates from Thoong-Heang Village (TH), 12 isolates from Na-Ngam Village (NN), and one isolate from Lao PDR. Twenty two singletons are obtained as shown in smallest circles; 10 singletons (Haplotype B, D, E, F, G, H, I, J, K and L) from Na-Yao Village (NY), seven singletons (Haplotype N, O, P, Q, R, S, and T) from Na-Ngam Village and five singletons (V, W, Y Z and A1) from Thoong-Heang Village. The reference isolates from Thailand and Lao PDR are grouped in Haplotype B1 and C1. (B) The haplotype network of 31 haplotypes using *nad1* sequences. Haplotype G presents the highest frequency of 9 isolates; 7 isolates from Na-Ngam Village, one isolate from Na-Yao Village and one isolate from Lao PDR. Twenty singletons are demonstrated; seven singletons (Haplotype B, C, D, E, F, H and I) from Na-Yao Village, three singletons (Haplotype I, K, M and N) from Thoong-Heang Village, four singletons (Haplotype O, P R and S) from Na-Ngam Village, five singletons (Haplotype T, U, V, Y and Z) from Vietnam and one singletons (Haplotype E1) isolate from Lao PDR. The reference isolates from Thailand, Lao PDR, Vietnam and Cambodia are clustered in Haplotype X, A1, B1, C1 and D1. Each open circle represents each haplotype and the numbers of each haplotype were placed in parentheses. Each singleton is marked with the names for villages and isolates. The solid line shows the network relationship of the haplotypes. The frequency of nucleotide change between haplotypes is shown as black bullets.

The haplotype network of 29 haplotypes from 69 *cox1* sequences from three villages and the reference isolates revealed relative haplotypes in the populations of *O*. *viverrini*. Haplotype M, which presented the highest frequency of isolates, was dominant and contained 20 isolates; 12 isolates from Na-Ngam Village, seven from Thoong-Heang Village and one isolate from Lao PDR (JF739555.1). However, 22 isolates were determined to be singleton; Haplotypes B, D, E, F, G, H, I, J, K, L, N, O, P, Q, R, S, T, V, W, Y, Z and A1. Moreover, nine singletons (Haplotypes D, E, F, G, H, I, J, K and L) from Na-Yao Village showed a distinct genetic difference from the other haplotypes. Additionally, the reference isolates from Thailand and Lao PDR were grouped as Haplotypes B1 and C1 (Fig 3A).

The network of 31 haplotypes from 67 *nad1* sequences showed closely related haplotypes. Haplotype G had the highest frequency of isolates consisting of 9 isolates; 7 isolates from Na-Ngam Village, one isolate from Na-Yao Village and one isolate from Lao PDR (JF739555.1). The *nad1* haplotype network revealed 20 singletons (Haplotypes B, C, D, E, F, H, I, K, M, N, O, P, R, S, T, U, V, Y, Z and E2). Two haplotypes (E and F) from Na-Yao Village and five haplotypes (J, K, L, M and N) from Thoong-Heang Village demonstrated the genetic diversity from the other haplotypes. Moreover, reference isolates from Thailand, Lao PDR, Vietnam and Cambodia were clustered in 11 haplotypes (T, U, V, W, X, Y, Z, A1, B1, D1 and E1). From the *cox1* and *nad1* haplotype networks, *O*. *viverrini*, isolated from Na-Yao and Thoong-Heang Villages, showed a higher genetic difference than those from Na-Ngam Village or reference isolates (Fig 3B).

### Phylogenetic trees based on the *cox1* and *nad1* sequences of *O*. *viverrini*

**Fig 4:**
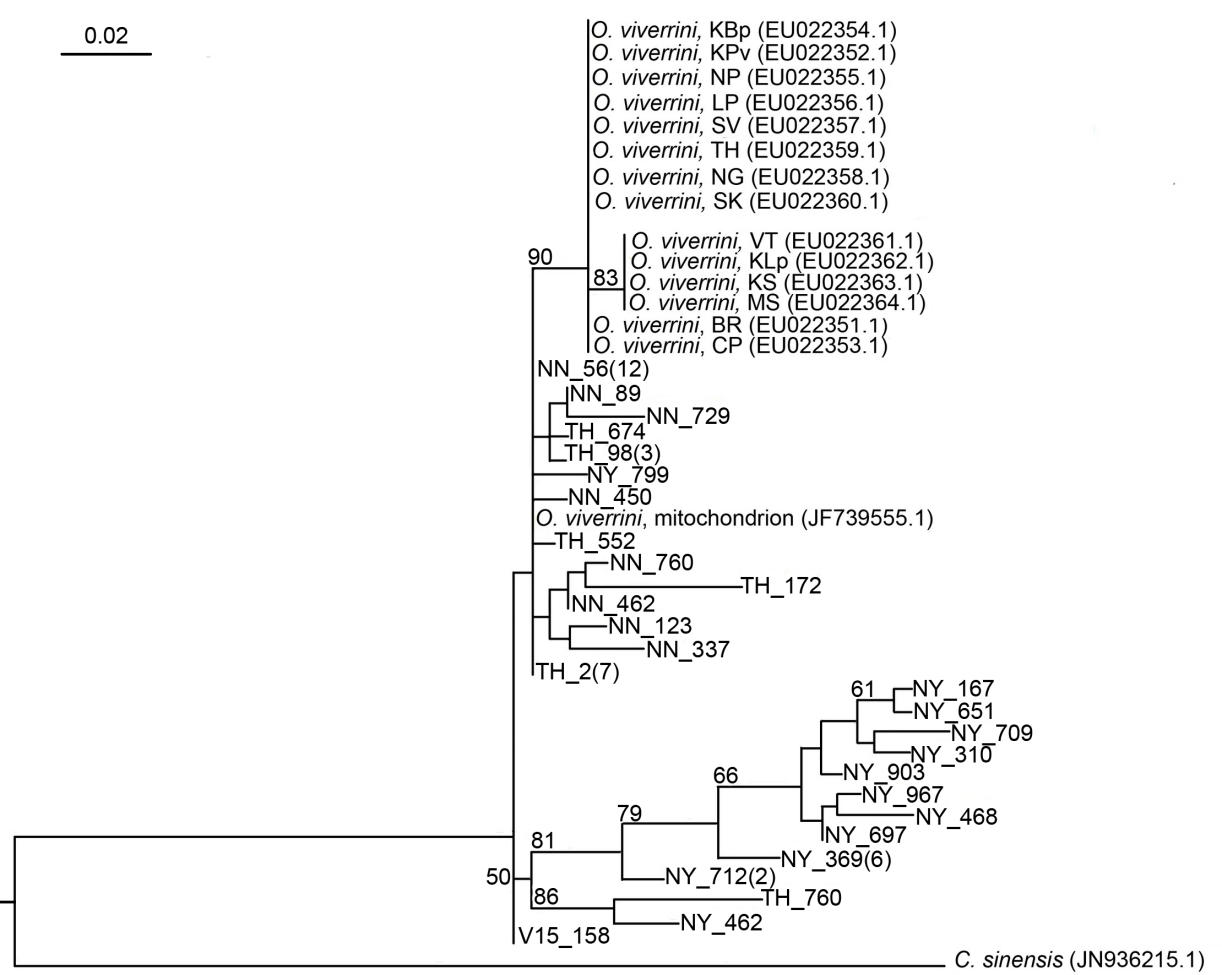
The Randomized Axelerated Maximum Likelihood tree based on the *cox1* sequences of *O*. *viverrini*. The alignment of 393 nucleotide sequences without gaps and 27 variants from three villages (NY = Na-Yao, NN = Na-Ngam, TH = Thoong-Heang) were analyzed. *O*. *viverrini* Thai isolates (CP, BR, KBp, KPv, SK, NG, MS, KLp, KS) and Lao PDR isolates (SV, VT, *O*. *viverrini* mitochondrion partial genome) were used as the reference isolates. The percentages of 1,000 replications (bootstraps) of more than 50% are shown at the nodes. The number of samples is in parentheses.

**Fig 5:**
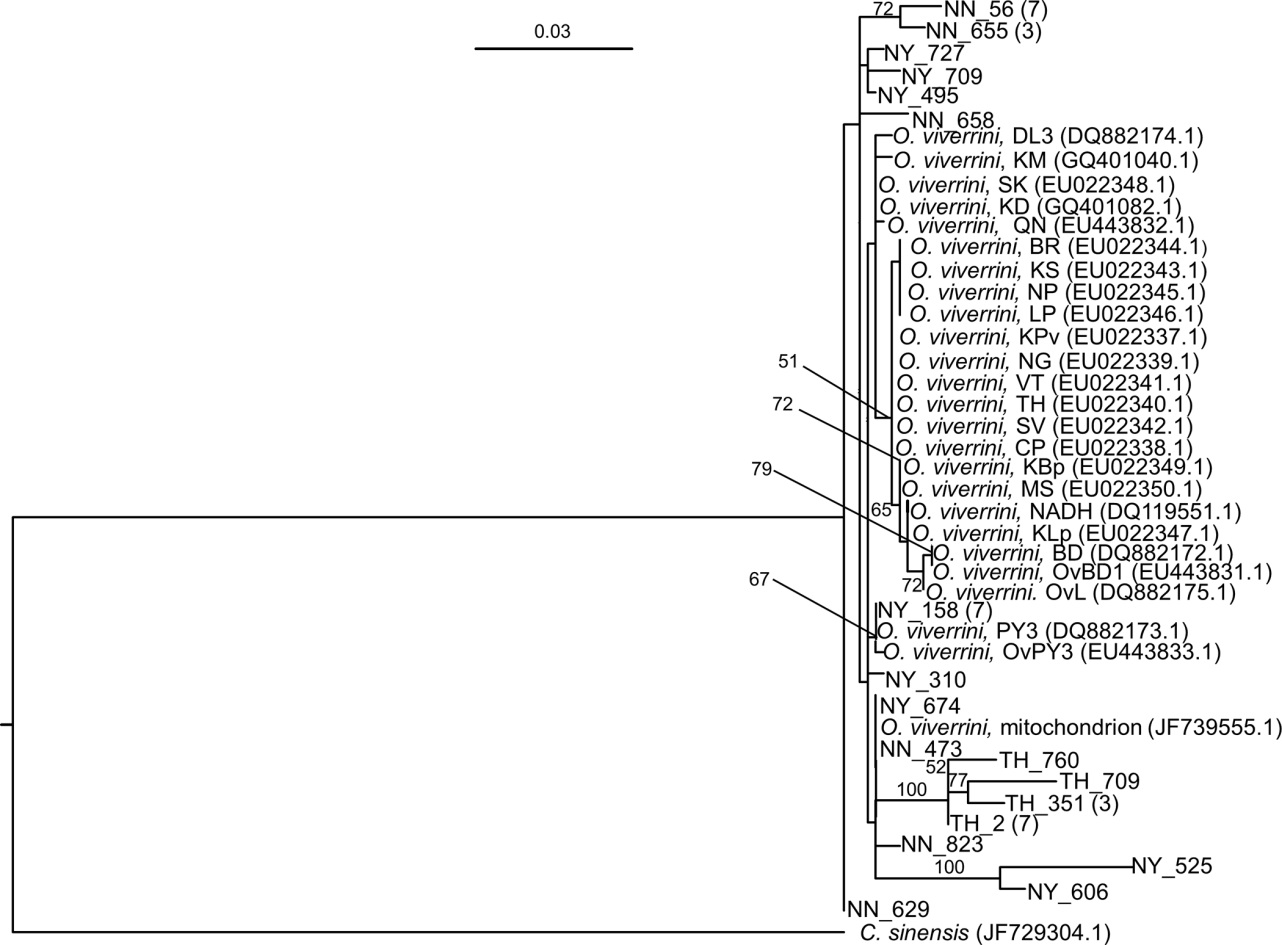
The Randomized Axelerated Maximum Likelihood tree based on the *nad1* sequences of *O*. *viverrini*. The alignment of 688 nucleotide sequences without gaps and 18 variants from three villages (NY = Na-Yao, NN = Na-Ngam, TH = Thoong-Heang) were analyzed. *O*. *viverrini* Thai isolates (BR, KS, LP, NP, KPv, MS, KLp, KBp, SK, NADH1), Lao PDR isolates (CP, NG, SV, VT, KM, *O*. *viverrini* mitochondrion partial genome), Vietnamese isolates (BD, OvBD1, OvL, PY3, OvPY3, QN, DL3) and a Cambodian isolate (KD) were used as the reference isolates. The percentages of 1,000 replications (bootstraps) of more than 50% are shown at the nodes. The number of samples is in parentheses.

The list of selected *cox1* and *nad1* reference sequences used in the RAxML tree construction is shown in Table 1. Figs 4 and 5 illustrate the phylogenetic relationships among of *O*. *viverrini* generated by RAxML based on 393 and 668 bp of the *cox1* and *nad1*, respectively. The tree topologies of two genes, *cox1* and *nad1*, showed concordance that reference isolates of *O*. *viverrini* were mostly clustered in monophyletic group with high bootstrap value (90%) in *cox1* (Fig 4) but low (<50%) in *nad1* (Fig 5), whereas *O*. *viverrini* in the study showed to be paraphyletic to the reference isolates. Low bootstrap value (<50%) demonstrates potentially shift in divergent order causing uncertain diversity among our *O*. *viverrini* in *nad1* tree but not in *cox1* tree. In addition, two clades containing 13 isolates from Thoong-Heang Village and two isolates from Na-Yao Village were separated by strong bootstrap values to support 100% (Fig 5). Moreover, *O*. *viverrini* in central Thailand is clearly distinct from common *O*. *viverrini* found within Southeast Asia, i.e., northeastern Thailand, Lao PDR, Vietnam and Cambodia because there is no *O*. *viverrini* of central Thailand grouping in the same reference cluster as the commonly found *O*. *viverrini* (Figs 4 and 5).

## Discussion

The overall *O*. *viverrini* infection from PCR-RFLP profiles among the three villages was 9.6% which was higher than the average prevalence (3.8%) reported in central Thailand [44]. However, positive identification of *O*. *viverrini* might have been underestimated because the sensitivity of ITS2-PCR assay was only 84.9% and 141 positive fecal samples were unavailable for molecular analysis. In this study, especially in Na-Ngam Village, using microscopic examination, the prevalence of *Opisthorchis*-like eggs infection was 25.9%, the highest prevalence among the three villages. Related studies have reported the prevalence of *O*. *viverrini* infection was 21.3% conducted in Na-Yao Village, Sanamchaikate District in 2009 [42] and was 24.0% in 2013 in the same area [43]. Independent risk factors associated with *O*. *viverrini* infection included age over 60 years and consuming fresh raw fish salad [42,43]. The predominant FBT infection found in this study was *O*. *viverrini* infection (9.6%), confirming Sanamchaikate District as one of the endemic area of *O*. *viverrini* infection in central Thailand. Although public health interventions for FBT are available in the area *i*.*e*., access to treatment for infected people, re-infection with FBT is very common due to the unchanged eating habits of the rural elderly. A foundation to prevent and control *O*. *viverrini* infection should focus on improving knowledge of disease severity, reducing risk habits of becoming infected, treating infected individuals as well as promoting self-awareness of people in the community.

Using the RTFluke primers, the sensitivity of the PCR obtained from this study was higher when compared with 71.0% sensitivity performed in one related report [24]. Although most fecal samples obtained for this study revealed light infection of *Opisthorchis*-like eggs (Eggs per gram of feces <1,000), we used a combination technique of breaking up the eggs using freeze-thawing plus PBS incubation to increase the efficiency of DNA extraction [24,28]. However, some fecal samples were unavailable for DNA extraction and PCR assays due to an insufficient amount of feces for the PBS ethyl acetate concentration technique. Thus, discrimination results of those samples could not be obtained. The negative PCR assay results might have been due to strong PCR inhibitors in fecal samples and unsuccessful breaking up of the eggs. Thus, improvements in DNA extraction as well as the PCR assay to detect *Opisthorchis*-like eggs in fecal samples are still required, particularly for those with only a light infection.

Interestingly, using PCR-RFLP analysis of ITS2 region, a related cross-sectional study conducted in Na-Yao Village in 2009 firstly revealed *C*. *sinensis* infection among Thai villagers. Eggs and adults of *C*. *sinensis* were recovered from stool samples [24]. The presence of *C*. *sinensis* is well known in northern part of Vietnam and northern including southern parts of China. However, only one report of human *C*. *sinensis* infection was found in central Thailand [24]. The result urged us to explore the real situation of *C*. *sinensis* infection in Sanamchaikate District. Of the 253 samples positive for *Opisthorchis*-like eggs, none were identified as eggs of *C*. *sinensis* using the ITS2-PCR assay [24]. As a result, at present, *O*. *viverrini* is the only human liver fluke identified in fecal samples of villagers. A related report of *C*. *sinensis* infection in this area in 2009 could be an uncommon situation when a group of people acquired *C*. *sinensis* infection [24]. The specific time and exact location of consuming the infected uncooked fish contaminated with *C*. *sinensis* metacercariae could not be determined. Those infected fish may therefore have been brought from outside the country. Unfortunately, in-depth interviews of the sources of food consumption from those infected people were not performed at that time. Thus, from our negative results of *C*. *sinensis* eggs using the ITS2-PCR assay of *Opisthorchis*-like eggs, persistent *C*. *sinensis* infection in this community has not been evidently proved. To confirm the *C*. *sinensis* life cycle in the study area, the infected snail intermediate host, *Melanoides tuberculata*, as well as infected cyprinoid fish with metacercariae need to be observed.

PCR-RFLP profiles in this study revealed not only *O*. *viverrini* infection, but mixed infection of *O*. *viverrini* and *H*. *taichui* were also observed. The PCR products of *E*. *albuferensis* showed the same size as *H*. *taichui* and similar agarose gel results; therefore, DNA sequencing was required for species identification. Regarding the non-comparative number of *O*. *viverrini* infections, the prevalence of both *H*. *taichui* and *E*. *albuferensis* were less than 1%. In our study, stool examination revealed operculated trematode eggs of 90 to 120 μm in size, similar to those of the medium intestinal trematodes. The ITS2-PCR assay and nucleotide sequencing confirmed human *E*. *albuferensis* infection in a central community of Thailand. However, little is known about the pathology of *E*. *albuferensis* in human infection since one report of *Euparyphium* sp. infection was first described in Lao PDR in 2012 [53]. To the best of our knowledge, *E*. *albuferensis* is a trematode in the family Echinostomaidae, the medium intestinal trematode, of which freshwater snails are the first and second intermediate hosts in the life cycle. The first snail intermediate host is *Gyraulus chinensis*, while various kinds of snails (*Lymnaea truncatula*, *L*. *peregra*, *L*. *palustris* and *Physa acuta*) could serve as a second intermediate host [54]. Thus, the consumption of raw or uncooked freshwater snails is the source of infection. *H*. *taichui*, is a small intestinal fluke, commonly reported in many parts of Thailand, where the highest prevalence has been reported in northern Thailand [55,56]. However, none of the related studies have confirmed *H*. *taichui* infection in central Thailand. Our study revealed two infections of FBT, predominantly *O*. *viverrini* and a minority of *H*. *taichui*, identified from *Opisthorchis*–like eggs in the study area.

This study is the first to reveal the genetic structure of *O*. *viverrini* in central Thailand. The neutrality tests were used to determine evolutionary changes at molecular level and genetic differentiation between species and without significance using the *cox1* and *nad1*. Sequences analysis of *O*. *viverrini* eggs using Fu’s *Fs* test revealed no population expansion and population bottleneck of *O*. *viverrini* in this community. Together with Tajima’s *D* test of the *cox1* and *nad1* sequences, no statistical significance of the test could confirm a lack of population size expansion and lack of decrease in population size or balancing selection. As a result, the populations of *O*. *viverrini* obtained from the three villages were defined as monophyly. Elderly individuals in Sanamchaikate District originally moved from the northeastern region; thus, they could have harbored *O*. *viverrini* and transmitted it to the area. Our results also agreed with the previous study that the populations of *O*. *viverrini* along the Mekong River including Thailand, Lao PDR and Cambodia were monophyly using *nad1* sequence analysis [40]. The haplotype networks and RAxML phylogenetic analysis of the *cox1* and *nad1* sequences indicated that *O*. *viverrini* isolated from Na-Yao and Thoong-Heang Villages revealed genetic differences from the reference isolates [30]. Haplotype M from *cox1* and Haplotype G from *nad1* were extremely frequent and contained most isolates from Na-Ngam Village and Lao PDR (JF739555.1), indicating that they were possibly an older haplotype than the singletons and widely distributed throughout this community. In addition, *O*. *viverrini* nucleotide sequences obtained from this study were not clustered with reference isolates except *O*. *viverrini* (JF739555.1) from Lao PDR; therefore, it was probably the original isolate of *O*. *viverrini* in this community. However, no significant differences of genetic diversity were observed within and among *O*. *viverrini* populations. Our results supported the previous studies stating that *nad1* is a powerful molecular marker to study genetic relationships of *O*. *viverrini* [39,40]. This study has shown the diversity of *O*. *viverrini* in central Thailand that could potentially be an isolated population, whereas factors of genetic diversity are unclear. Thus, more studies of the genetic diversity of *O*. *viverrini* in this endemic area should be conducted using other powerful genetic markers as well as to study a number of samples collected from other communities of nearby provinces. Moreover, using a single *O*. *viverrini* egg-PCR assays for studying genetic structure could significantly increase the reliability of the test.

In conclusion, the ITS2-PCR assay has proved useful for detecting and characterizing *Opisthorchis*-like eggs. Our study confirmed that the endemic area of opisthorchiasis is still remained in Sanamchaikate District, a rural community of Chachoengsao Province, central Thailand. The genetic structure of *O*. *viverrini* in this study area is more closely related to *O*. *viverrini* from Lao PDR than to species from northeastern Thailand.
